# Rethinking Complex Care Using Participatory Medical Cognition and User-Driven Learning Amidst Multimorbidity: Participatory Action Research Study

**DOI:** 10.2196/81950

**Published:** 2025-12-09

**Authors:** Rahul Kulkarni, Mansi Agrawal, Tamoghna Biswas, Madhava Sai Sivapuram, Braahmani Bobba, Nivedita Pal, Sailaja Gade, Rishika Koloti, Abdul Salam, Ayushi Tandon, Champakali Biswas, Aditya Samitinjay Gade, Rakesh Biswas

**Affiliations:** 1 Co-founder, DoNew, Pune, India; 2 Mahatma Gandhi Memorial Medical College, Indore, India; 3 Freelance Consultant, Pediatric Hepatologist, Kolkata, India; 4 School of Public Health, Faculty of Health and Medical Sciences, The University of Adelaide, South Australia, Adelaide, Australia; 5 Kamineni Institute of Medical Sciences, Sreepuram, Narketpally, Nalgonda District, Telangana State, Nalgonda, Hyderabad, 508254, India, 91 9121046928; 6 Patient Advocate, PaJR, West Bengal, India; 7 Independent Researcher, Hyderabad, India; 8 Independent researcher, Pune, India; 9 Duhok university, Duhok, Iraq; 10 Trinity Business School, Trinity College Dublin, Dublin, Ireland; 11 Consultant Ophthalmologist, Kolkata, India; 12 Specialty Trainee, Department of Endocrinology and Diabetes, Sycamore House, Watford General Hospital, Watford, England, United Kingdom

**Keywords:** participatory medicine, case review, participatory cognition, patient engagement, user driven healthcare, patient journey records, PaJR

## Abstract

**Background:**

Managing patients with multiple chronic comorbidities is complex and challenging within traditional health care systems due to the need for multidisciplinary expertise, longitudinal tracking, and coordination. The development of collaborative online platforms leveraging user-driven health care (UDHC) and medical cognition principles offers new avenues for addressing these complexities by facilitating remote, participatory, and evidence-informed case management.

**Objective:**

The aim of this study was to demonstrate the application of a collaborative online case-based blended learning ecosystem (CBBLE) integrated with a patient journey record (PaJR) for the comprehensive remote management and review of a complex patient case with multiple chronic conditions. The study also aimed to evaluate how participatory medical cognition through this platform supports decision-making, patient empowerment, and clinical outcomes in a resource-constrained rural setting.

**Methods:**

A single case study of a 44-year-old female patient from rural India with multiple chronic conditions—including type 2 diabetes mellitus, Meesmann corneal epithelial dystrophy postphototherapeutic keratectomy, recurrent infections, lateral epicondylalgia, and hypertension—was managed remotely from December 2024 to May 2025. Deidentified health data, patient-reported outcomes, biometric monitoring, images, and historical records were shared asynchronously via an online e-log book platform. A global community of multidisciplinary experts engaged in collaborative review, critical evidence appraisal (including artificial intelligence [AI]–assisted literature retrieval), and ongoing clinical discussions. The patient advocate facilitated detailed symptoms and lifestyle logging. This case is intended to illustrate feasibility rather than establish generalizability.

**Results:**

The participatory platform enabled multispecialty expert input and integrated patient context to optimize management. The patient reduced antidiabetic medication significantly and discontinued all blood pressure and heart rate medications by March 2025. Lifestyle modifications, muscle-strengthening exercises, and diet adjustments were effectively supported. Expert consensus reclassified her irregular heart rate symptoms as anxiety-related palpitations, safely withdrawing beta-blockers. Collaborative discussions guided conservative management of eye infections and pain syndromes. Despite ongoing challenges with some symptoms (eg, eye issues and arm function), the patient reported improved quality of life, confidence, and satisfaction from reduced medication burden and comprehensive monitoring.

**Conclusions:**

This case exemplifies the value of collaborative, multidisciplinary, and technology-enabled participatory medical cognition platforms for managing complex multimorbidity. By integrating patient-reported data, AI-supported evidence synthesis, and asynchronous expert consultation, such ecosystems can enable holistic, evidence-based care, reduce overtreatment, support patient empowerment, and enhance clinical education, particularly in resource-limited and geographically dispersed contexts. Although this study presents a single case, wider adoption of similar digital platforms could significantly improve management of complex patients and foster a new model of user-driven, participatory health care and learning.

## Introduction

Patients presenting with multiple, interacting chronic conditions represent a growing challenge in modern medicine [1]. Effective management often requires integrating expertise from various specialties and adapting treatment plans based on the patient’s real-world experiences and responses, which can be difficult to coordinate in traditional, fragmented health care settings [2]. The need for improved approaches to navigate diagnostic and therapeutic uncertainty in complex cases is paramount [3].

The evolution of the internet, particularly the transition from Web 1.0 (a passive library) to Web 2.0 (an interactive forum allowing user participation) and beyond, has paved the way for new models of health care interaction. Concepts such as user-driven health care (UDHC) [4,5], where multiple health care stakeholders, including patients, interact online to understand and make decisions, and “medical cognition” [6] encompassing various human and artificial cognitive tools to resolve clinical complexity, are emerging. These approaches aim to leverage collective intelligence and asynchronous communication to improve patient care.

From a relational perspective, records of patient journeys are to be understood not as static carriers of information, but as a dynamic flow of information that shapes and is influenced by users’ actions, technology availability, institutional settings, and cultural norms [7]. This also opens up the possibility for learning from dialogic interactions, where patient journey records, health care providers, and clinical learners co-construct meaning over time across various contexts.

In this article, we demonstrate how our case-based blended learning ecosystem (CBBLE) combines online and offline components for case-based learning and integrates patient journey records (PaJRs) to facilitate participatory medical cognition [8]. The PaJR focuses on patient-reported outcomes, allowing us to learn from the patient’s journey beyond the prescribed treatment and the evolving decisions made during the phases of illness along the course of his life [9]. These platforms not only provide material resources but also actively structure collaborative interactions, adapting to sociocultural contexts and supporting learning in cases of complex multimorbidity. Drawing upon the concept of relational affordances [9,10], the PaJR-CBBLE system serves not merely as a digital repository but as a communication scaffold that enables training in human medical cognition. We illustrate this participatory approach through the management of a 44-year-old female patient with diverse and fluctuating chronic conditions, drawing upon the detailed record of discussions, interventions, and outcomes captured within the online platform. Similar to challenges highlighted in other contexts, such as the ethical and clinical dilemmas of overused medicine and the lack of shared decision-making in interventions like percutaneous coronary intervention (PCI) [11], this case presented its own complexities requiring careful, evidence-informed management. A previous study by Podder et al [8] demonstrated the potential of CBBLE in reducing overdiagnosis and overtreatment by fostering evidence-based, patient-centered inputs through coordinated team communication. This aligns with the patient’s expressed gratitude for achieving health improvements “without much medication and without getting expensive tests” that were previously common but yielded no improvement. The aim of our study was to understand and explain collaboration around PaJR workflow not just as information sharing, but as a dialogic and socio-material process [12,13], where we contribute toward a more nuanced understanding of how PaJRs can serve as a communication device in CBBLE for training of medical cognition [14,15] for managing complex multimorbidity. Unlike conventional EMR-driven systems that primarily support clinician documentation, the PaJR-CBBLE model brings patients and artificial intelligence (AI) tools directly into the collaborative reasoning process.

## Methods

### Instrumental Details

The collaborative online medical case review took place within an online e-log book platform [16] designed for discussing deidentified patient health data with a global online community of experts. The primary objective was to find solutions to the patient’s clinical problems using collective, current best evidence-based inputs via our platform, which exemplifies a participatory setting within user-driven health care.

**Figure 1.**
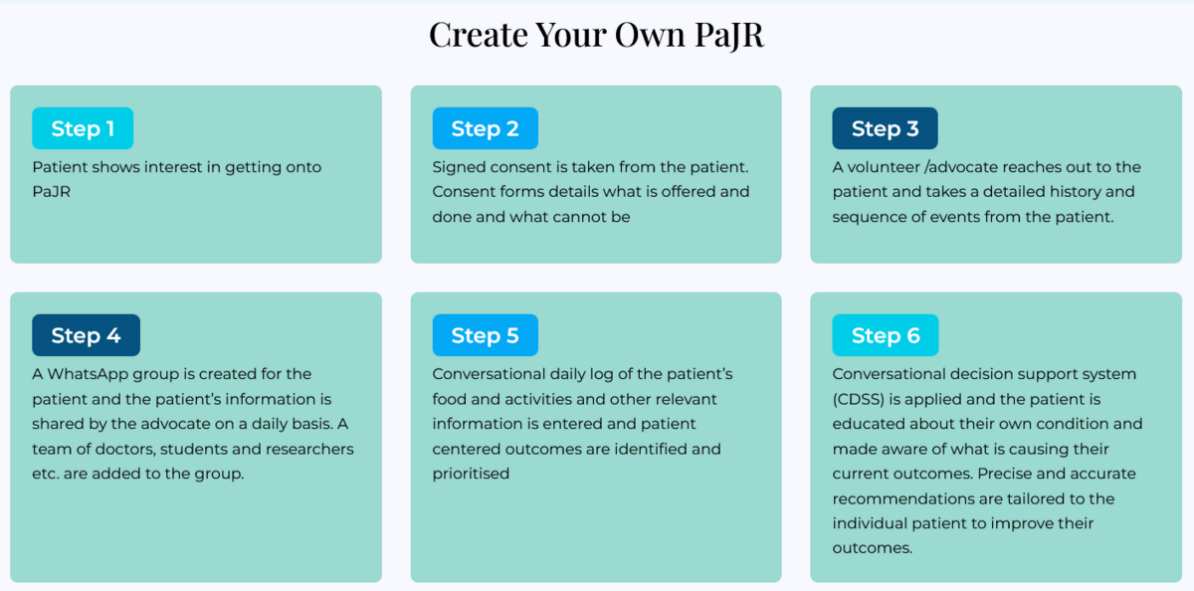
Steps involved in creating the patient journey record (PaJR). (Source: pajr.in).

The platform used several techniques inherent to user-driven health care and participatory medical cognition. Below, we present an abstracted actant and activity (both human and nonhuman) view of this process. We highlight the relevant affordances [17,18], agencies, and materialities that became relevant as we make various agential cuts [9,19] in the process described in Figure 1. Several techniques and resources were used in the development of patient journey records (see Textbox 1).

Textbox 1. **Techniques and resources used in patient journey records (PaJRs).**

- Collaborative online platform: an asynchronous online e-log book served as the central hub for communication and documentation. This provided a persistent, asynchronous space for patient case documentation and collaborative feedback. It also enabled distributed communication and longitudinal dialogue between stakeholders, decoupled from temporal and spatial constraints.
- Deidentified data sharing: patient privacy was protected by sharing deidentified patient documents, images, and health data. Signed informed consent was obtained for this. This allowed shared access to patient data for collaborative diagnostic interpretation and opened up spaces for distant experts to ethically enter the interpretive dialogue without compromising privacy.
- Patient-reported outcomes: the patient advocate (PA) served as the primary liaison, sharing detailed subjective accounts of the patient’s symptoms, pain levels, daily activities, meals, and general well-being, providing crucial context. This is a key component of the PaJR system. Ongoing reporting by the PA fosters cointerpretation and helps the patient move from being a passive recipient to an epistemic contributor [20]. The PaJR workflow encourages patients or their primary caregivers to become their own advocates while maintaining deidentified interactions. Therefore, the PA can be either the patient himself/herself or be a patient’s guardian, identity remaining unknown to the working team while working through the virtual communication system. Group discussion participants in the online PaJR cannot identify the patient unless they have previously met face-to-face. This approach protects anonymity while still allowing the patient’s voice, through or alongside the PA, to be integral to the case record. All data, including symptom logs, biometric measurements, and images (such as meal and eye photos), were uploaded directly by the patient advocate into the online platform. The PA shared detailed subjective accounts of the patient’s symptoms, pain levels, daily activities, meals, and general well-being, providing crucial context. This continuous logging in the case used for this study allowed the patient to “pinpoint what food intake is affecting her blood glucose levels” and to “understand how her blood sugar levels were throughout the day and correlate her energy levels” by sharing hourly activity. The patient’s historical medication record revealed that nearly 8‐10 years ago, she was on antidiabetic medications (metformin 850 mg after breakfast and a combination of metformin 500 mg and glimepiride 1 mg after dinner). The dosage was significantly reduced during the current intervention period. As part of the detailed history captured, the patient reported use of propranolol 10 mg and bisoprolol 2.5 mg from 2018 onward for irregular heart rate symptoms. These medications had been discontinued for 5 months by the time of this report, and group experts interpreted these symptoms as likely palpitations associated with anxiety, rather than a formal arrhythmia diagnosis.

Regular monitoring and logging: the PA reported objective measurements including blood sugar readings and blood pressure measurements over time. These were logged consistently to help identify trends and inform decisions.

- Regular monitoring and logging: the PA reported objective measurements, including blood sugar readings and blood pressure measurements, over time. These were logged consistently to help identify trends and inform decisions.
- Sharing and reviewing images/documents: visual data, such as eye photos and meal photos, and historical medical records were shared and reviewed by experts. Images (eyes and meals), past records act as visual genres enabling richer multimodal dialogue and expert interpretation.
- Expert consultation and discussion: experts who are primary PaJR monitors (PPMs), included from various specialties internal medicine (PPMs), physiatry (PPM 3), and ophthalmology (ophthalmologist / ophthalmic expert 2), provided specialized consultation, debated approaches, and synthesized information.
- Evidence review and critique: experts discussed and referenced external medical literature and AI-synthesized information to inform decisions. This included a critical evaluation of the quality and limitations of available evidence. Experts compared AI-suggested findings with real-world data, applying a careful epistemic judgment. This process draws upon System 2 thinking [21], making communication and thinking slower for analysis.
- AI Integration for information and translation: throughout the patient’s journey within the online e-log book, AI tools played a significant role in facilitating information retrieval and synthesis, especially as part of the collaborative, evidence-based approach. AI tools operate as external cognitive scaffolds, helping participating clinicians and even the patient quickly access medical information and summarize knowledge landscapes. Meta AI (Meta Platforms, Inc), Perplexity (Perplexity AI, Inc), and ScholarGPT (Sider.AI) were regularly consulted by team members to quickly access and summarize medical information. AI tools were used as part of the case, not as final authorities, but as “dialogic partners” providing competing interpretations, generated ideas, and reference points that clinicians evaluated to better inform their own decisions [22,23]. Details of the specific AI tools and versions used (ChatGPT-4.1 [OpenAI], ScholarGPT by Sider.ai 2025, Meta AI WhatsApp 2025, NotebookLM by Google 2025) and their comparative use cases are described in the section “Use Cases and Effectiveness of AI Tools in Clinical Cognition and Communication.”
- Structured case report (PaJR) system: a structured case report was maintained to document the patient's journey, discussions, and findings for reference and learning. The case report became an index of dialogic activity [12,13], pattern recognition, and medical judgment, while also serving as an output contributing to broader knowledge dissemination and education. This process was acknowledged by the patient, who sought clarification on how publication would open up new avenues for medical treatment and education, highlighting its role in spreading knowledge within the scientific community.

The cast of characters (Information S1 in Multimedia Appendix 1) involved included the patient advocate (PA), various physician participants (PPMs 1, 3, 4, 5, 6, 7) with distinct roles from lead clinician or coordinator (PPM 1) to specialists like a physiatrist (PPM 3) and team members involved in admissions and monitoring, a case reporter (CR) responsible for the PaJR, ophthalmology experts, other PAs contributing personal insights (eg, 33F PA), artificial intelligence tools (Meta AI, Perplexity), and the patient herself, whose data and experiences were central to the discussion.

Timeline: the case discussion and management activities documented in the sources spanned from December 2024 to May 2025 (Information S2 in Multimedia Appendix 1).

### Case Description

#### Overview

The patient’s journey, documented through the online platform, revealed the dynamic nature of her multiple chronic conditions and the team’s collaborative efforts to manage them. The timeline summary provides a structured overview of symptoms, reports, discussion, interventions, and results. The affordances described above reflect the outcomes of decisions and actions taken by various human and nonhuman participants throughout the ongoing, interactive process of working with the PaJR’s participatory medical cognition device. They are in no way definitive, but they played an important role in managing the clinical condition of a patient’s case and learning from the patient’s journey with the flow as described below.

#### Pre-December 2024

The patient had a history of wisdom teeth issues, leading to infection and antibiotic treatment. She was diagnosed with type 2 diabetes mellitus, experiencing fatigue, weakness, abdominal cramps, appetite loss, difficulty eating, early satiety, and a sour taste. She also had hypertension and a gallbladder issue. Notably, she reported recurrent low-grade fever and significant weight loss (10 kg in 2 months) before December 2024. Her long-standing history included corneal dystrophy diagnosed in 2018, causing severe eye pain, photophobia, itching, and redness until her phototherapeutic keratectomy (PTK) in 2018. She had experienced blurred vision since 2017 and dry eyes since childhood, using eye drops from the age of 16 years. Other symptoms included headache, migraine, vomiting tendency, and recurrent subconjunctival hemorrhage with high fever starting at age 19. Interventions prior to the online platform included dental treatment, antibiotics, diabetes medication (initially reducing blood glucose to 174 mg/dL), PTK, admissions, and long-term use of eye drops. Blurred vision persisted post-PTK.

#### December 2024

The patient presented with giddiness, which worsened after taking oral contrast for a planned computed tomography (CT) abdomen for a potential gastric or duodenal diverticulum. Sphincter of Oddi dysfunction (Type 2) was suspected based on Rome II criteria for biliary pain [24]. Monitoring of blood pressure (BP; 2 hourly) and blood sugars (fasting, 2 hours postmeal) was initiated, along with informed consent collection for the case report. The antidiabetic regimen was adjusted, first to glimepiride 1 mg before breakfast and metformin 500 mg after lunch, then revised to metformin 500 mg after every meal in addition to glimepiride 1 mg before breakfast. The CT abdomen was performed with oral contrast and appeared normal, as did the gallbladder and pancreas, aligning with previous normal serum amylase and lipase. The patient’s general random blood sugar (GRBS) was 86 mg/dl and BP was 110/70 mm Hg.

#### January 2025

The patient experienced abdominal cramps and indigestion after eating nuts and sprouts. Advice included sharing food plate images and following the Harvard plate proportion [25], continuing metformin after every meal. Amidst complaints of weakness, headache, dizziness, and eyes feeling dark, the team advised hourly BP readings and adjusted Bisoprolol dose. BP and sugar monitoring continued, showing fluctuating readings.

#### February 2025

The headache continued despite feeling generally better. Episodes of low BP occurred (85/59 mm Hg supine, 80/50 mm Hg standing). Advice included walking short distances. Recurrent low-grade fever (99.4-100 F) with stomach discomfort and painful, stiff hand muscles resumed. Pain and stiffness in the tennis elbow area (lateral epicondylalgia) worsened with activity and improved with rest or a band. A physiatrist expert was added to the group for activity modification suggestions [26]. Advice included using the hand "palm up" and reducing the glimepiride dose, with continued sugar monitoring. BP readings remained low sometimes. Requests were made for historical ophthalmic documents regarding corneal dystrophy.

#### March 2025

Issues with the sugar monitor were reported, suggesting switching to continuous glucose monitoring (CGM) and requesting specific sugar readings. Discussions requested deidentified lateral arm and abdomen images for the case report to document phenotype, noting that maintaining muscle and losing abdominal fat could help reverse diabetes. The patient reported weight and waist measurements and shared photos. Fluctuating upper abdominal pain, discomfort, feverish feeling, choked throat, cough, and chest congestion occurred. Symptoms were suspected to be due to GERD, with advice on elevating the head of the bed. Despite the fever subsiding, body aches, headache, and severe cough persisted. Symptomatic paracetamol and steam inhalation were advised for the cough. The potential relationship between throat pain, inflammation, and hyperglycemia was noted and substantiated using Meta AI. An eye issue resembling episcleritis was noted. Ophthalmologist’s clinical findings from a local evaluation were shared, confirming left corneal opacity with epithelial changes (likely Meesmann dystrophy post PTK) and right corneal epithelial changes with an iris Lisch nodule, suggesting Meesmann and ruling out neurofibromatosis Type 1. Diagnosis of Meesmann corneal epithelial dystrophy was confirmed based on clinical workup. The patient is status post-PTK performed in 2018 for symptomatic relief. An ophthalmologist’s opinion stated the corneal dystrophy was not very significant on the current exam, but pre-PTK visual acuity data were needed. After detailed monitoring during the March 2025 follow-up, the BP medication was stopped entirely [27]. In addition, the patient disclosed that she had been self-administering propranolol 10 mg and bisoprolol 2.5 mg (since 2018 for “irregular heart rate.” Upon collaborative review, this was interpreted as probable anxiety-related palpitations rather than a documented arrhythmia. These medications had been stopped for approximately 5 months at the time of reporting without adverse cardiovascular events. The patient has remained off BP medicine since then. Symptoms of weakness, darkness of eyes, and pain in the shoulder and arms recurred. Discrepancies between the patient’s BP machine and ward machines were observed, leading to checks with multiple machines. Discrepancies were also noted between the patient’s glucometer and ward readings.

#### April 2025

Severe hair fall was reported. Androgen excess was suggested as a possible explanation. Discussion occurred regarding checking vitamin D levels and critically reviewing evidence and guidelines for vitamin D deficiency in the Indian context [28], highlighting potential artifacts from using Western cut-offs and assay variations. AI tools were discussed in the context of evaluating hair loss. Efforts continued to obtain pre-PTK visual acuity data, but the file was reported missing. A summary and recommendations regarding the corneal dystrophy post-PTK were provided, emphasizing that PTK is for symptomatic relief rather than acuity, and management should focus on dry eye, refractive correction, and monitoring. Severe hair fall was reported. The patient believed this increased hair loss from December to April was due to the frequent antibiotics she was consuming during that time. Despite adherence to advice, the patient identified persistent issues where the platform “did not aid in relief” in certain instances, such as recurring eye problems and her inability to straighten her arm despite following instructions.

#### May 2025

The patient reported right-hand muscle weakness and neck pain. The patient was learning muscle-strengthening exercises. Ophthalmic opinion suggested oral antibiotics for a likely internal hordeolum, noting diabetic patients' proneness to recurrent infection. The efficacy of antibiotics for hordeolum and chalazion was debated based on available evidence accessed via Web 2.0, [29] which suggested doubtful benefit over warm compresses and hygiene. Fexofenadine was noted as helpful for itching. A detailed management plan for hordeolum was provided, emphasizing warm compresses, eyelid hygiene, and reserving antibiotics for complications or failure after 1 week. A topical antibiotic ointment was also suggested. Oral antibiotics (azithromycin and amoxicillin-clavulanate) were recommended by PPM 4. While a 10 kg weight loss was noted before December 2024, the patient further reported a clinically significant loss of “more than 8 kgs in 6 months” (December 2024 to June 2025) which she attributed to her adherence to the recommended diet and muscle strengthening exercises. Discussion continued about the role of surgery (incision and curettage, steroid injection) for resistant styes after 4-6 weeks of failed conservative treatment. The distinction between internal and external hordeolum and management implications was discussed. Therapeutic uncertainty regarding oral doxycycline for hordeolum was noted, reiterating the lack of high-quality evidence and reliance on lower-level evidence and pathophysiological rationale. Practical recommendations emphasized the stepwise approach starting with conservative measures. This discussion highlighted the challenge of relying on lower-level evidence and pathophysiological rationale when high-quality trials are absent, a feature of Web 3.0 medical cognition. Table 1 summarizes 5 chronic conditions of the patient based on diagnostic timeline, interventions suggested, monitoring, and the current status of the patient.

**Table 1.** Patient’s chronic and acute conditions summary.

Condition	Diagnostic date	Key interventions	Monitoring	Status
Type 2 diabetes mellitus	Pre-December 2024	Metformin 500 mg after meals; glimepiride 1 mg before breakfast (later reduced); dietary changes (Harvard plate); symptom tracking. Previously on higher doses of antidiabetic medications of metformin 850 mg after breakfast and a combination of metformin 500 mg and glimepiride 1 mg after dinner a decade ago. These were reduced significantly during the current care process	Blood glucose (fasting, postmeal, and random); GRBS^a^ readings; symptoms (fatigue, weakness, and dizziness)	Currently on 750 mg/day Metformin only; glimepiride and BP^b^ medicines fully withdrawn since March 2025. Glucose is under better control with lifestyle corrections and regular monitoring
Meesmann corneal epithelial dystrophy (S/P PTK)	2018	Phototherapeutic keratectomy (PTK; 2018) long-term use of lubricating eye drops; ongoing management focused on dry eye and refractive correction	Ophthalmic evaluations; observation of epithelial changes	Persistent dry eye symptoms status post-PTK; currently not visually significant; regular ophthalmic review advised
Lateral epicondylalgia (tennis elbow)	February 2025 (worsening)	Activity modification; physiatrist input; positioning advice (“palm up”)	Subjective pain response; functional limitation tracking	Intermittent worsening; improving with rest and ergonomics; physiatric management ongoing
Internal hordeolum (eye infection)	May 2025	Warm compresses (first line); eyelid hygiene; topical antibiotic ointment; oral antibiotics debated (azithromycin or amoxicillin-clavulanate if not improving); steroid and surgical options discussed for resistant cases	Symptom resolution (pain, swelling, and redness); response to conservative therapy	Likely internal hordeolum diagnosed; conservative measures initiated; oral antibiotics used selectively; under observation
Hypertension	Pre-December 2024	Bisoprolol (dose adjusted); later stopped by patient temporarily	2-hourly BP monitoring; standing and supine BP readings; multiple machine checks due to device discrepancies	BP medicine (bisoprolol) was completely stopped in March 2025 and has remained off since then, with regular monitoring

a GRBS: general random blood sugar.

b BP: blood pressure.

### Ethical Considerations

This manuscript describes a single patient journey as a part of an overarching project on clinical complexity for which appropriate clearance has been obtained from the institutional ethics committee of Kamineni Institute of Medical Sciences, Narketpalli, Hyderabad [30]. Written informed consent by the patient was taken before initiation and submission of this manuscript. The study and case review were explained to the patient in the local language. All the potential data, such as images, were deidentified to ensure the confidentiality of the patient and the PA. Consent was also obtained before PaJRs were formed by all the patients showing interest in joining the PaJR technology in their lifestyle and disease management. No financial or other compensation was provided to the patient for participation.

## Results

The case presented above demonstrates the application and advantages of a collaborative online platform grounded in the principles of medical cognition and UDHC. This participatory setting, acting as an online e-log book and a component of a CBBLE, enabled remote, multidisciplinary management of a patient with multiple comorbidities (diabetes, corneal dystrophy, recurrent infections, and pain syndromes). In the instrumental details section, we discuss various affordances around PaJR that became relevant as the patient’s care unfolded as a dynamic process involving ongoing interpretations and decision-making shaped by material tools and dialogic exchanges.

The platform facilitated input from various specialists at one place (physiatry, multiple ophthalmologists, general physicians, and PPMs), which is often challenging in traditional clinical set-ups, overburdening the patient to keep up with multiple follow-ups from different departments and extra-logistical work. This was crucial in navigating diagnostic nuances, such as distinguishing between potential episcleritis and hordeolum, or understanding the implications of Meesmann corneal epithelial dystrophy in a patient status post-PTK. For example, the patient’s historical use of heart rate, modulating drugs for perceived arrhythmia was reinterpreted as somatic anxiety rather than arrhythmia, prompting appropriate discontinuation.

The platform fostered collective evidence review and synthesis. Experts actively discussed and critiqued medical literature, reflecting a core aspect of evidence-based medicine. The detailed debate surrounding the efficacy of antibiotics for hordeolum, referencing published literature and discussing the hierarchy of evidence, is a prime example. PPM 5’s comment regarding the need to accept different levels of evidence in the context of Web 3.0 medical cognition highlights the practical challenges and adaptive thinking required when high-quality randomized controlled trial evidence is lacking. Also, the clinical learners (health care providers) participating (observing or engaging) in this process are encouraged to embody the system 2 thinking inherent in medical cognition, facilitating slower, more analytical processing of information.

While EMR systems also enable storage and sharing of patient data among physicians, they are typically oriented toward documentation rather than dialogic interaction. By contrast, the PaJR platform extends beyond a clinician-EMR dyad by integrating patients, PAs, and multidisciplinary experts into a single asynchronous workspace. The addition of AI tools further differentiates PaJR, not as a passive repository, but as an active cognitive partner that supports evidence retrieval, synthesis, and translation within ongoing case discussions. This combination, PaJR+ doctor+patient+AI, creates a participatory and iterative process that conventional EMR-based collaboration does not provide.

Notably, the platform’s role is here not constrained to data logging; it facilitates participatory distributed medical cognition [31] through discussion, dissent, and iterative reinterpretation. We noted that participants were also actively involved in discussions, and all the questions and inputs were facilitated in a conversational approach, in contrast to the usual medical learning and teaching pattern, which often involves long didactic lectures. Therefore, this aspect aligns with user-driven learning and the creation of user-driven learning community ontologies, which weave contextual patient data into a tapestry for reasoning and AI-driven processing and allow participatory learning for medical students. We are calling this participatory medical cognition.

This was powerfully echoed by the patient, who expressed being “extremely happy” with reduced medication and regained confidence, stating “I’ve gotten back a much better life” and “I can now do my own work.” This positive sentiment underscores the platform’s capacity to facilitate holistic care. This reduction is particularly striking given that when she was on antidiabetic medications with metformin 850 mg after breakfast and a combination of metformin 500 mg and glimepiride 1 mg after dinner a decade ago. Through the platform’s participatory care approach, she now takes only 750 mg Metformin daily and has stopped both glimepiride and BP medicine entirely since March 2025. This was possible only because of the patient’s commitment to battling her sarcopenia and trunkal obesity through regular sharing of her muscle strengthening exercises from the gym, as seen in the images logged on April 30 in the 44-year-old female case’s PaJR [32] and her well-regulated diet, as seen in the images of food plates in the 44-year-old female case’s PaJR [32]. This demonstrates the role of continuous lifestyle-guided care, intensive monitoring, and informed stepwise withdrawal of medication. At the same time, data logging enabled the patient to critique care gaps, where the clinical collaboration either empowered self-care (particularly weight loss, diet, and exercise) or did not offer symptom relief (arm-straightening and eye complaints), showing both the empowering and limiting dimensions of participatory care. Progressive reduction of blood pressure and anti-diabetic medications can be seen in Multimedia Appendix 1.

The integration of technology and AI was evident. AI tools like Meta AI and Perplexity were used for rapid information retrieval and synthesis on specific topics, such as the link between inflammation and hyperglycemia or evidence for antibiotic use. While human expert validation remained crucial, AI served as a tool to augment the collective information-gathering process. This aligns with the use of asynchronous intelligence AI tools in human cognition, where continued reliance on Web 3.0 tools (Meta AI, Perplexity, and large language models) is integrated as a dialogic supplement for medial cognition (another participant), not a replacement, to clinical judgment.

While not explicitly framed within a formal shared decision-making (SDM) model as discussed in the context of PCI, the process inherently involves shared information and facilitates a degree of patient involvement (through the PA) in understanding options and rationale, potentially leading to more confident choices. The continuous logging of patient data by the PA allowed the team to identify patterns, understand potential triggers (diet, travel, and weather), and observe the interplay between conditions. Discrepancies noted between the patient’s self-monitored BP and sugar readings and ward measurements prompted collaborative troubleshooting and investigation into potential device issues or measurement techniques. This was instrumental in the patient’s experience and to a certain extent, the emergence of her own medical cognition (or health cognition), as she felt “monitored very closely which helps her adhere to a good lifestyle” and found the group provided “accountability especially with regard to food intake.” The platform also has emergent affordances for the PA. The PA, acting as the liaison, was empowered to share detailed patient information and receive collective expert advice relatively quickly. This participatory approach also provided the patient with “mental strength and a sense of hope that she is not alone,” enabling her to “ask and update about her symptoms in the group everyday.” She also found motivation to “make innovative healthy food for herself”’ and became “‘more conscious about outside food.”

The CR’s role in deidentification and case report maintenance was vital to this process. The patient’s direct query about the impact of publishing her case report “‘will this open up new avenues.. for students?”’ and the assurance that it would “‘spread further in the scientific community”’ to help “‘many more patients”’ clearly demonstrates the platform’s role in fostering user-driven learning and community ontologies.

Overall, we find that maintenance of a structured case report via the PaJR system also serves as documentation and a learning resource. The PaJR captures the patient’s journey, discussions, and findings, contributing to a broader “participatory medical cognition” and serving as an educational tool for participants. The timeline in a case report represents more than a linear record; it embodies ongoing negotiations of meaning where patient experiences, clinical interpretations, and treatment decisions are continuously shaped by social and technological interactions within the platform.

We looked for patients connected via PaJR for similar outcomes. Interestingly, we noted another such case where a 63-year-old male with metabolic syndrome, dyspnea and previously diagnosed hypertension was able to stop his antihypertensive therapy with regular BP recordings shared on his PaJR journey by engaging with some clinicians as part of this study. It was argued, and doctors concluded, that BP recordings were not consistently high enough to merit further antihypertensive therapy [33]. This suggests the current clinical practices observed in different parts of the world, where prescription of antihypertensives based on single clinic BP recordings could be leading to the over-diagnosis of hypertension. Thus, we can argue on the basis of another PaJR that clinical judgment and decisions when made using active participatory data sharing approaches can help ensure optimal treatment plan, preventing over-diagnosis following over-treatment. Table 2 shows the specific uses of AI in clinical cognition and communication.

**Table 2.** Specific uses of artificial intelligence (AI) for this patient’s management and in the patient journey record (PaJR).

Tool	Use case	Type of use	Reception/impact	Effectiveness (perceived by stakeholder participating in clinical care via the PaJR)
Meta AI	Explanation of otoliths and vertigo	Definition of medical terms; explanation of physiology	Provided educational content; acknowledged by group	Effective
Meta AI	Inflammation and hyperglycemia link	Scientific substantiation; literature-backed explanation	Supported patient advocate’s observation; received positively	Effective
Meta AI	Food allergies	List of allergens; risk guidance	Directly influenced dietary decision-making	Effective
ChatGPT	Translation of diet plan to Bangla	Translation (English to Bangla)	Patient advocate clarified feasibility of advice	Useful, though advice needed contextual adjustment
ScholarGPT	Diagnostic uncertainty in hordeolum	Detailed analysis; differential diagnosis; literature synthesis	Highly valued by medical expert	Highly effective
ScholarGPT	Pinpointing past conversations	Retrieval of relevant past case discussions	Experts expressed strong approval	Highly effective
ScholarGPT	Summary and recommendations	Summarization; structured recommendations	Acknowledged with appreciation	Highly effective
Meta AI	Postprandial blood sugar testing	Definition; guidance based on guidelines	Integrated into discussion; clarified by clinician	Effective
ScholarGPT, ChatGPT	Vitamin D levels analysis	Concise overview and deep dive; synthesis of literature	Appreciated and endorsed by group	Highly effective
ScholarGPT, ChatGPT	AI in hair counting and singularity	Technical exploration; potential future application	Acknowledged with appreciation	Effective
Perplexity	Doxycycline for hordeolum	Evidence review; therapeutic clarification	Prompted reflection on evidence hierarchy	Effective
August AI	Calorie counting	Nutritional analysis of meals from images	Used by advocate to guide dietary choices	Useful
ChatGPT	Mermaid.js code generation	Creation of structural diagram code	Used for paper figures	Effective
NotebookLM	Paper writing support	Synthesis of sources for academic writing	Used in background for drafting	Effective

## Discussion

### Theoretical Contribution

Theoretically, we identify that the digital platform functions as a generative socio-material infrastructure [12,13,34] not merely as a passive data repository, but as an active participant in the shaping of health care practice and cognition. This active participation that is emerging is coming together of social and technical entities with multiple materialities: patient-generated data, AI tools, biometric monitoring devices, historical clinical records, images, and expert annotations. These material entities, each with their own material properties [17,18], interact relationally to enable clinical sensemaking as part of participatory medical cognition. By allowing the capture of evolving symptomatology, monitoring data, expert discussions, and therapeutic interventions in a structured, but nonlinear, timeline, the platform enables the asynchronous construction and reconstruction of the patient’s health narrative. Thus, the PaJR becomes more than a sequential log; it is a dialogic space where diverse voices (patient, PA, clinicians, and AI tools) negotiate meaning, context, and care pathway. The interventions and decisions documented on the platform, ranging from medication adjustments to nuanced symptom analysis, illustrate habitual and situated usage of the emergent technology capabilities available in the context to the users. These uses reflect an epistemic practice whereby digital technologies support medical cognition training [14,15]. Learners engaging with this data-rich environment are not only exposed to complex case material but are also trained to manage diagnostic uncertainty, connect multimodal clinical inputs, and critically assess contextualized evidence over time.

Through repetition, reflection, and feedback, the platform (PaJR) thus serves as an online participatory medical cognitive apprenticeship space, enabling both clinicians and patients to participate in a dialogic and deeply contextual model of clinical reasoning aligned with the complexities of multimorbidity.

### Relevance for Practice: Use Cases and Effectiveness of AI Tools in Clinical Cognition and Communication

Description of our case revealed that during the course of collaborative case discussions, a variety of AI tools were used to support both clinical reasoning and patient communication. All tools referenced were those current and available in 2024‐2025. This included ChatGPT 4.1 (OpenAI), ScholarGPT (Sider.ai, 2025 version), Meta AI (WhatsApp integration, 2025 release), Perplexity Deep Research (2025), and NotebookLM (Google, 2025). These tools were used for translation (eg, diet plan translation into Bangla, official language in Indian state, for patient use), definition of medical terms (eg, explaining otoliths and their role in vertigo), evidence retrieval (eg, substantiating the link between inflammation and hyperglycemia, or evaluating doxycycline use in hordeolum), and diagnostic support (eg, differentiating internal from external hordeolum). They also helped with summarization, retrieval of past conversations, generation of structured recommendations, and practical tasks such as calorie estimation from food images or producing diagram code for case figures. The range of applications shows how AI extended beyond simple fact-finding to include contextual translation, data synthesis, and structured knowledge support for both clinicians and patients. This integration of AI allowed for comprehensive information gathering and contributed to the platform’s role as an educational tool by highlighting where robust evidence was lacking.

Each AI tool was used for different tasks. Meta AI served as a quick reference for definitions and clinical guidelines. ChatGPT was used for practical applications like language translation and creating structural diagrams. For deeper reasoning, the team relied on ScholarGPT and Scholar ChatGPT**,** which helped with evidence review and drafting treatment recommendations. Perplexity was used for specific therapeutic questions, while August AI provided instant calorie and nutrient information. NotebookLM was used for synthesizing information to help with the paper’s writing. The group found that the usefulness of each AI depended on matching the right tool to the specific task.

### Directions for Future Work

This single-patient case study demonstrates feasibility but does not establish generalizability. Several directions for future research emerge from the limitations identified. First, expanding to pilot cohorts or multi-case series will help test the consistency of outcomes and enable broader learning from diverse patient journeys. Larger studies with standardized data collection will also permit systematic evaluation of clinical impact. Second, controlled before-and-after or comparative study designs will be essential to rigorously assess whether AI-expert collaboration improves decision-making and outcomes compared with usual care. Third, the opportunistic use of multiple AI tools in this case highlights the need for structured comparisons to identify which tools provide the most value in supporting collaborative cognition. Furthermore, the majority of AI use cases were presented to stakeholders, including patients, who were able to challenge and contribute by either validating or refuting the AI-created outputs [35]. Future research should therefore compare the implications of using varied AI tools from the patient’s perspective and investigate potential governing mechanisms from their viewpoint [36]. Fourth, we recommend research on contextually relevant platform features, such as referral and transfer mechanisms for conditions that cannot be resolved remotely, as well as hybrid models combining local examinations with remote monitoring. Finally, researchers can investigate strategies to sustain patient and their advocate engagement in daily logging, such as reminders, gamification, or community reinforcement, will be critical for ensuring data completeness in rural or resource-constrained settings. Collectively, these directions will allow this model to move from proof-of-concept toward scalable, evidence-based participatory medical cognition ecosystems.

### Limitations

Some challenges remain evident in our case too, such as ensuring complete historical documentation and the inability to replace face-to-face physical examination for certain conditions was common. The patient’s feedback provides specific examples, noting instances where she “did not get satisfying answers or solutions to her problems” regarding “recurring eye problems” and her inability to “straighten her arm.” Hybrid models combining remote monitoring with periodic local examinations could help address this gap, particularly in rural or resource-constrained contexts. Moreover, since sustained daily logging depends on ongoing patient and advocate motivation, future designs should incorporate supportive strategies such as reminders, gamification, or community reinforcement to promote long-term adherence.

In addition, the case underscores the importance of integrating a referral or transfer mechanism within such platforms. Certain conditions, such as refractory eye symptoms or musculoskeletal limitations, may require in-person evaluation. Future iterations of this system should embed structured referral pathways and follow-up modules to ensure timely escalation to local or tertiary care when remote management is insufficient. Although our system enables participatory medicine, the daily logging of activities is still dependent on the patient and PA’s interest to continue further.

Compared to previous literature on overuse of PCI [11], this case offers complementary insights into participatory decision-making and resource optimization. While the financial burden or invasive procedure concerns seen in PCI were absent here, the case underscores the shared need for diagnostic clarity and judicious use of interventions [25]. The patient’s appreciation for improving without excessive tests or medications affirms the platform’s role in promoting prudent care. Similar to the CBBLE in earlier work, this platform united multidisciplinary inputs and patient-reported outcomes. However, unlike direct physician-patient interactions in PCI cases, the asynchronous, proxy-mediated communication posed unique challenges for real-time shared decision-making. To offset the limits of asynchronous communication, future deployments could incorporate secure synchronous modalities—such as scheduled teleconsultations or large language model–assisted phone triage through medical students—while still maintaining the persistent advantages of an asynchronous record.

### Conclusion

The case study of the current patient and their PaJR effectively illustrates the potential of collaborative online platforms rooted in user-driven health care and medical cognition principles for managing complex patients with multiple chronic conditions. It is not just about managing a patient with multiple illnesses; it is about seeing the digital platform itself as a generative socio-material infrastructure. For instance, the study highlights a shift in the roles of patients and clinicians. The platform transforms the patient from a passive recipient of care into an epistemic contributor, actively shaping their own treatment and knowledge. The patient’s direct experience validated this generative potential of the digital platform, as she reported increased confidence, improved daily functioning, and satisfaction with reduced medication requirements, enhancing her overall quality of life. The participatory nature of the digital platform allowed that AI tools were used, not as final authorities, but as “dialogic partners” providing competing interpretations, generated ideas, and reference points that clinicians evaluated to better inform their own decisions. Overall, we find that the digital platform as a participatory setting supported evidence-based decision-making and provided an educational ecosystem for health care stakeholders. While challenges related to data collection consistency and the limitations of remote physical assessment persisted, the advantages demonstrated in this case suggest that similar online platforms can significantly enhance comprehensive, patient-centered care, particularly in resource-constrained or geographically dispersed settings. Further development and widespread adoption of participatory digital platforms, integrated into case-based blended learning ecosystems, hold promise for improving outcomes and fostering a more collaborative learning and evidence-informed practice of medicine.

## Supplementary material

10.2196/81950 Multimedia Appendix 1 Deidentified chronological data of the case with specific focus on decisions made to manage multiple comorbidities.

## Abbreviations

AI: artificial intelligence
CBBLE: case-based blended learning ecosystem
CGM: continuous glucose monitoring
CR: case reporter
CT: computed tomography
GRBS: general random blood sugar
PA: patient advocate
PaJR: patient journey record
PCI: percutaneous coronary intervention
PTK: phototherapeutic keratectomy
SDM: shared decision-making
