# Supplementary material for: Rethinking Complex Care Using Participatory Medical Cognition and User-Driven Learning Amidst Multimorbidity: Participatory Action Research Study
Source: J Particip Med. 2025 Dec 9;17:e81950. doi: 10.2196/81950 (PMC12688023; doi:10.2196/81950)
Supplement: Multimedia Appendix 1 [file jopm-v17-e81950-s001.docx]

### **Information S1**

### **Cast of characters and roles played**

Based on the provided sources and our conversation history, the interaction takes place within an **Online E Log Book**, which serves as a platform for discussing a patient's de-identified health data with a global online community of experts. The goal is to find solutions to the patient's clinical problems using collective current best evidence-based inputs.

Here is a summary of the cast of characters, their roles, the techniques they used, and the effect of their interactions:

**Cast of Characters and Their Roles:**

- **Patient Advocate (PA):** This individual acts as the **primary liaison** between the patient and the expert group. The PA shares detailed updates on the patient's symptoms, daily activities, meals, blood sugar readings, and blood pressure measurements. They convey the patient's history, ask questions seeking advice and clarification from the experts, and relay expert instructions and advice to the patient. The PA is also responsible for sharing de-identified patient documents and images or facilitating their deidentification.
- **PPM 1:** Appears to be a **lead clinician or coordinator** of the discussion. They initiate and guide the conversation, assign tasks to other team members (PPMs and CR), provide medical opinions and advice, review patient data, and integrate information from various sources, including AI tools and literature. PPM 1 also uses the handle "cm" in some conversations.
- **PPM 3:** Identified as a **Physiatrist**. They are involved in monitoring patient vitals like blood sugar and BP, provide expertise related to activity modification, contribute to discussions on research and evidence, particularly regarding Vitamin D, and summarize medical concepts and management plans, including providing translations.
- **PPM 4:** Participates in patient admissions, facilitates medical imaging like CT abdomen, is involved in patient reviews, monitors vital signs, requests access to patient reports, and provides specific recommendations for eye conditions, including medication and diagnostic steps.
- **PPM 5:** Involved in managing patient reports, including handling privacy concerns. Also part of the patient review team and contributes to discussions on evidence.
- **PPM 6 & PPM 7:** Involved in implementing monitoring orders in the ward and managing patient consent forms.
- **CR (Case Reporter):** Responsible for the **creation and maintenance of the patient's case report (PaJR)**. They assist with de-identifying patient documents for sharing.
- **Ophthalmologist / Ophthal expert 2:** These roles represent **Ophthalmology experts** who provide specialized consultation on the patient's complex eye conditions, including corneal dystrophy (Meesmann's) and eyelid issues (hordeolum/chalazion). They interpret clinical findings and images, discuss treatment history (PTK), evaluate management strategies, and differentiate between conditions. "S" and "pm" appear to be handles used by these experts.
- **Meta AI & Perplexity:** **Artificial intelligence tools** consulted by the team for specific information retrieval and synthesis on medical topics like otoliths and vertigo, food allergies, and evidence regarding antibiotics for hordeolum.
- **33F PA:** Another **Patient Advocate** who contributes insights based on their own or others' experiences with similar problems, specifically related to hair loss and Vitamin D.
- **Patient:** The individual whose complex health conditions and experiences are the central focus of the discussion. Her history, symptoms, objective measurements, and response to treatment drive the conversation and expert inputs.

**Techniques Used:**

- **Collaborative Online Platform:** Utilizing an online E Log Book for asynchronous communication and documentation.
- **De-identified Data Sharing:** Protecting patient privacy while allowing experts to review relevant information.
- **Patient-Reported Outcomes (PROs):** The PA frequently shares detailed subjective accounts of the patient's symptoms, pain levels, general well-being, and daily experiences, providing crucial context for the objective data.
- **Regular Monitoring and Logging:** Tracking vital signs (BP, sugar) and activities over time to identify patterns and evaluate interventions.
- **Sharing and Reviewing Images/Documents:** Utilizing visual data (CT scans, eye photos, meal photos) and historical medical records to aid diagnosis and management discussions.
- **Expert Consultation and Discussion:** Experts across different specialties (general medicine, physiatry, ophthalmology) provide opinions, debate approaches, and synthesize information.
- **Evidence Review and Critique:** Experts discuss and reference external medical literature and AI-synthesized information to inform decisions. This includes discussing the quality and limitations of available evidence.
- **Utilizing AI for Information and Translation:** Integrating outputs from Meta AI and Perplexity to quickly access information and attempt language translation.
- **Case Report (PaJR) System:** Documenting the patient's journey, discussions, and findings in a structured format for future reference and learning.

**Effect of Interactions and Techniques:**

- **Comprehensive Care Management:** The collaborative approach allowed for the management of a patient with multiple co-morbidities (Diabetes, Corneal Dystrophy, etc.) by drawing on diverse expertise remotely.
- **Timely Advice and Reassurance:** The patient, through the PA, received relatively quick responses and advice during symptomatic periods, including during episodes of fever, pain, or eye discomfort.
- **Detailed Patient Understanding:** The continuous logging of patient data, activities, and symptoms allowed the team to gain deeper insights into potential triggers (like diet spikes, travel, weather changes, or even sun exposure) and the interplay between her conditions (e.g., throat pain/infection and sugar levels, diet and abdominal cramps/sugar).
- **Evidence-Based Decision Making:** The discussions frequently referenced available evidence and guidelines, leading to advice aligned with current medical understanding, while also highlighting areas where robust evidence is lacking (e.g., antibiotics for hordeolum).
- **Educational Platform:** The detailed discussions, evidence reviews, and critiques served as an educational tool for the participants, including understanding the nuances of conditions like Vitamin D deficiency in specific populations or the management of eyelid infections. The PaJR served as both documentation and a learning resource. The patient's direct query about the impact of publishing her case report – 'will this open up new avenues... for students?' – and the assurance that it would 'spread further in the scientific community' to help 'many more patients' clearly demonstrate the platform's role in fostering user-driven learning and community ontologies.
- **Identification of Discrepancies:** The comparison of patient's self-monitored BP with ward measurements highlighted potential issues with devices or measurement techniques.
- **Integration of AI:** The use of AI tools demonstrated their potential for rapidly summarizing research and providing information, although human expert interpretation and validation remain crucial.
- **Challenges Highlighted:** The process also revealed challenges inherent in remote collaboration, such as obtaining complete historical documents, ensuring consistent data collection (e.g., BP measurement), and the difficulty of replacing face-to-face physical examination for certain conditions.

Patient Feedback

The patient, a 44-year-old female from rural India, provided comprehensive feedback on her experience with the collaborative online platform and its impact on her health and quality of life. This feedback was crucial for understanding the direct benefits and limitations of the participatory medicine approach from a user's perspective.

- Overall Satisfaction and Empowerment: The patient expressed being "extremely happy" with the care received, noting that her blood pressure medication was stopped (though it had been temporarily stopped before) and her diabetes medicine dose had been reduced. She reported a significant improvement in her life, stating, "I’ve gained confidence, and I’ve gotten back a much better life" where she can now "do my own work". She felt deeply grateful for achieving health improvements "without much medication and without getting expensive tests" that were previously common but yielded no improvement.
- Specific Positive Impacts and Learnings: The patient highlighted several measurable impacts attributable to the platform:
  - Constant contact with doctors and feeling "monitored very closely" which greatly aids her adherence to a healthy lifestyle.
  - The group provided "mental strength and a sense of hope that she is not alone", allowing her to "ask and update about her symptoms in the group everyday”.
  - She learned to "pinpoint what food intake is affecting her blood glucose levels" and to correlate her energy levels by sharing hourly activity.
  - The group fostered "accountability especially with regard to food intake".
  - She felt "seen and heard," which motivated her to "make innovative healthy food for herself" and become more conscious about outside food.
  - Her weight loss of "more than 8kgs in 6 months" (December 2024 to June 2025) was directly attributed to her adherence to the "recommended diet and muscle strengthening exercises".
  - She also noted that increased hair loss from December to April was believed to be "due to the frequent antibiotics she was consuming during that time".
- Patient's Engagement with AI Tools: Beyond the expert discussions, the patient herself actively engaged with digital tools, utilizing AI "once every 2-3 days when she tries out a new food recipe and doesn't know the amount of calories, protein and fat content in it". Her motivation to consistently update the group stemmed from feeling "accountable and empowered" and her "internal interest" in how the group assists her daily.

###

### **Information S2**

### **Timeline summary**

*Symptoms, reports, discussion, interventions, results*

Here is a summary of the patient's journey, symptoms, discussions, recommended and implemented interventions, and results, drawn from the provided sources along a timeline:

**Timeline Summary for the 44F Patient**

**Pre-December 2024:**

- **Symptoms:** Initial problems with **wisdom teeth**, leading to infection, itching, and requiring antibiotics. While on antibiotics, she experienced **fatigue and weakness**. She was found to be **diabetic** with a postprandial sugar level of 202 mg/dl. She also suffered from **cramps in the abdomen**. Starting August 2024, she lost appetite, felt weak, had difficulty eating, felt full quickly, health deteriorated, and experienced a **sour taste in the mouth**. She continued to have **no appetite** despite medication. In October, her **spo2 was 72**, **heart rate was high**, and she had a problem in her **gall bladder**. In the month prior to December 29, 2024, she had a **temperature of 99-100 F** every 2-3 days and lost **10 kgs of weight** in the two months prior. She was diagnosed with **corneal dystrophy in both eyes in 2018**. Symptoms in 2018 included **severe pain when opening eyes in the morning**, pain lasting all day, inability to go out during the day, **itching, redness, and sensitivity to lights**. These symptoms lasted until her PTK procedure. She experienced **blurred vision since 2017**, initially thinking it was due to diabetes. She faced day-to-day difficulties because of this. She had **dry eyes since childhood** and used eye drops since age 16. She also reported **headache, migraine, and vomiting tendency**. She experienced **blood hemorrhage in her eyes** (subconjunctival hemorrhage) with high fever, first noticing it at age 19. In October 2016, after losing her father and feeling depressed, she had **burning sensation in her eyes**, couldn't read sign boards, and had **watery eyes** when outside.
- **Interventions Implemented:** She was treated by a dentist with medication for wisdom teeth issues, then antibiotics for infection. She was given medication for controlling sugar levels. She had a PTK operation for corneal dystrophy in January 2024 on her left eye in 2018 at LVPEI. While admitted to a nursing home in West Bengal, she was prescribed several injections: Meropenem, Hiocimax, Pantodac, Drotin, Cyclopalm, and Ondam. She used eye drops since she was 16 years old.
- **Results:** Her sugar values initially came down to 174 mg/dl with medication. Her blurred vision symptoms persisted even after PTK.

**December 2024:**

- **Dec 29-30:**
  - **Symptoms:** Patient waiting at the OPD hall. Complains of **giddiness**. After taking oral contrast, she experiences **loose motion, nausea, feeling very weak, dry mouth, and feeling tasteless**.
  - **Discussions/Recommendations:** The case was discussed in an online E Log Book by global experts. Questioned why the patient wasn't admitted to the super speciality ward. Advised for **CT abdomen tomorrow morning** to look for gastric and duodenal diverticulum, requiring oral contrast. Discussed with radiology and surgery teams. Suspected to have **Type 2 Sphincter of Oddi dysfunction** based on Rome II criteria for biliary-type pain. Discussion about **biliary manometry** at NIMs/Srujan. Advised **monitoring BP 2 hourly** and **blood sugars (fasting and 2 hours post-meal) till Monday**. Advised collecting signed informed consent form. Antidiabetic schedule changed to **Glimepiride 1mg before breakfast** and **Metformin 500mg after lunch**. Later revised to **Glimepiride 1mg before breakfast** and **Metformin 500mg after breakfast, lunch, and dinner**.
  - **Interventions Implemented:** Patient went to room 78 in the OPD hall. Patient took oral contrast.
  - **Results:** CT abdomen done with oral contrast; noted as appearing normal. Gall bladder looks alright, pancreas mildly bulky but likely normal. Previous serum amylase and lipase were normal. Her GRBS was 86 mg/dl. BP was 110/70 mmHg.

**January 2025:**

- **Jan 4:**
  - **Symptoms:** Patient feels **abdomen cramp and indigestion** after eating nuts and sprouts. Symptoms started 30 minutes after eating nuts and sprouts: **pain and feeling fullness of belly**.
  - **Discussions/Recommendations:** Advised sharing images of food plates before eating. A diet plan link (Harvard plate proportion) was shared. Noted that the proportion of grains in a shared plate was more than fruits and vegetables; advised equal proportion. Advised to continue Metformin 500mg after every meal.
  - **Interventions Implemented:** Patient reported abdominal cramps after nuts and sprouts. Patient is vegetarian. Patient took nuts and sprouts at 8:30 am. Patient ate lunch at 11 am and dinner at 7 pm. Patient reported proportions of food items. Patient followed the recommended diet plan.
- **Jan 7:**
  - **Symptoms:** Since 2 days, feels **both side upper abdomen pain** after taking food and moving. Pain stays for a long time, starts 15-20 minutes after food, and continues for about 3 hours minimum. No pain on empty stomach. After breakfast feels **uneasiness in abdomen, vomiting tendency, acidity, and indigestion**.
  - **Discussions/Recommendations:** Noted that pain after food indicates slow intestinal movement. Advised to **take a walk for abdominal uneasiness**. Asked if she can take Dompan or others.
  - **Interventions Implemented:** Patient reported pain characteristics. Patient asked about medication for uneasiness/indigestion. Patient took only half cup veg soup for dinner on Jan 10.
- **Jan 12:**
  - **Symptoms:** Since yesterday evening, suffered with **vomitings** even after dinner.
  - **Results:** Feels a little better.
- **Jan 13:**
  - **Discussions/Recommendations:** Advised to share entire plate image and use a scale to estimate quantity.
  - **Interventions Implemented:** Patient shared image. Patient reported homemade ragi biscuits (no maida, atta, sugar).
- **Jan 19 (Sunday):**
  - **Symptoms:** Feeling **very weak and headache**. Headache started after 12 pm and continued. Feels **very uneasiness, dizziness, and eyes feeling dark**. Has been feeling dizziness and headache continuously since after 2 pm.
  - **Discussions/Recommendations:** Plan for Sunday: Fasting and 2-hour post-meal sugars, hourly BP readings. Asked about Bisoprolol dose. Can make Bisoprolol half from now on. For dizziness, **Phenargan 25 mg every 8 hours** recommended. For headache, **Paracetamol 650 every 6 hours for 1 day** recommended.
  - **Interventions Implemented:** Fasting sugar 118. 9 am BP 157/89, 2-hour post-lunch sugar 141, BP 124/52, Pulse 59. After tiffin (3 pm) took 1/2 tab Metformin, 2-hour sugar 130. 8:45 pm BP 105/48. At 7:45 pm BP 105/60, completed dinner, took Metformin 1/2 after dinner. Took Metformin 1/2 tab. Morning took bisoprolol 2.5 mg. 2 hours after dinner sugar 142.
- **Jan 20:**
  - **Symptoms:** Experiencing **dizziness and headache since yesterday**.
  - **Interventions Implemented:** Took Phenargan 25 mg every 8 hours for dizziness and Paracetamol 650 every 6 hours for 1 day for headache.
- **Jan 21:**
  - **Results:** BP readings: 117/76 (PR89), 109/66 (PR 80), 103/51 (PR 84), 103/40 (PR 86).
- **Jan 22:**
  - **Results:** BP 169/99 at 14:42, later resolved to 112/72.

**February 2025:**

- **Feb 1:**
  - **Symptoms:** Feeling better but **headache continuing**.
- **Feb 2:**
  - **Symptoms:** At 8 am, feels **very weak**. BP 85/59, PR 94. After 3 minutes of standing, BP was 80/50.
  - **Discussions/Recommendations:** Noted sugars are very well controlled.
  - **Interventions Implemented:** Fasting sugar 123. Before breakfast took Glimepiride 1mg. After breakfast took 1/2 tab Metformin 500. 2 hours after breakfast sugar 128. Lunch at 3 pm. After lunch took 1/2 tab Metformin 500. 2 hours after lunch sugar 92, later 104.
  - **Results:** Sugars were well controlled.
- **Feb 3:**
  - **Symptoms:** When riding in a car, auto, or bike, feels like **everything inside the stomach is shaking, and there's a feeling of dizziness**. This didn't happen before. During yoga, there's a lot of **pressure**. Feels **very weak, has no energy**, when BP is low, vision goes completely **dark and feels dizzy**. Sometimes mentions feeling a lot of **pressure in the chest**.
  - **Discussions/Recommendations:** Advised that for long distances, should get off a few kilometers earlier and walk.
- **Feb 8:**
  - **Symptoms:** Has had a **fever again since yesterday** (99.4-100 F), with a bit of **discomfort in the stomach**. Muscles in the **hand are quite painful and stiff**.
  - **Discussions/Recommendations:** Advised to check temperature every four hours, also check BP and sugar.
- **Feb 9:**
  - **Symptoms:** Stiffness in hand slightly improved with activity. Pain with **stiffness on her tennis elbow** (lateral epicondylolagia) since last 2-3 days. Pain gets worse when cleans room, carries weight, or cooks. Resolves with rest. Finds relief with Tennis elbow band.
  - **Discussions/Recommendations:** Noted that tennis elbow can resolve spontaneously, non-operative treatments help. Added a Physiatrist expert to the group for suggestions on activity modification. Advised using the hand with "palm up" for a couple of months. Reduction of **Glimepiride to 0.5mg from tomorrow** recommended, repeating sugars similarly next week.
  - **Interventions Implemented:** Fasting sugar 112 mg/dl, BP 116/78 mmHg. Before lunch took Glimepiride 1mg 1/2 tab. After lunch took Metformin 500mg 1/2 tab. 2 hours after lunch sugar 121mg/dl. After tiffin (3:05 pm) took Metformin 500mg 1/2 tab, 2 hours after tiffin sugar 112mg/dl. After dinner took Metformin 500mg 1/2 tab, post dinner sugar 90mg/dl. Patient reported specific activities aggravate pain and relief with rest/band.
- **Feb 10:**
  - **Interventions Implemented:** Took 0.5mg Glimepiride before meal and 250mg Metformin after meal.
  - **Results:** BP 90/60, after standing 92/58.
- **Feb 14:**
  - **Discussions/Recommendations:** Asked PA to share documents regarding corneal dystrophy, onset of visual symptoms, diagnosis date, previous symptoms, serial vision evaluation documents, and deidentified ophthal evaluations.
  - **Interventions Implemented:** PA confirmed diagnosis in 2018, described symptoms, confirmed blurred vision since 2017, described daily difficulties, confirmed dry eyes since childhood, use of eye drops, headache/migraine/vomiting history, and eye hemorrhage history.

**March 2025:**

- **Mar 2:**
  - **Symptoms:** Sugar monitor not working properly.
  - **Discussions/Recommendations:** Hoped monitor can be repaired; asked about age and warranty. Suggested switching to CGM. Asked for sugar at 9 PM and next day fasting and 2 hours after breakfast.
  - **Interventions Implemented:** Tiffin completed at 2:30 pm, took Metformin 500 in 1/2tab. Dinner at 7 pm.
  - **Results:** 2 hours after tiffin sugar level 234mg/dl. 2 hours after dinner sugar level 167mg/dl.
- **Mar 3:**
  - **Discussions/Recommendations:** Asked PA to share deidentified lateral view images of arm and abdomen for case report (phenotype description). Noted that if patient maintains her current phenotype (muscles in arm, abdominal fat), diabetes can be reversed easily. Noted weight loss is not always good (muscle loss), but paet loss (abdominal fat loss) is good and visible. Asked for previous lateral view photos for comparison.
  - **Interventions Implemented:** Fasting sugar 95mg/dl. Lunch with rice and cooked vegetables, took Metformin 500 1/2 tablet after lunch, post lunch sugar 126mg/dl. Ate puri, aloo sabji, apple, lime at 14:02 hrs. Patient reported weight 46kg, upper waist 30", middle portion 32". Patient shared previous lateral view photos.
  - **Results:** 2 hrs after tiffin sugar level 172mg/dl. This was noted as not high compared to other patients. Changes in phenotype were visible in shared photos.
- **Mar 7:**
  - **Symptoms:** After a gap of few weeks, has **upper abdominal pain**. Lot of **discomfort, couldn't sleep, felt feverish, throat completely choked, abdominal pain**.
  - **Results:** Feeling better later in the day.
- **Mar 8:**
  - **Symptoms:** **Severe cough developed, along with stomach, chest and head pain**. Nose blocked, chest congestion. Unable to sleep. Difficult when lying on bed, better sitting. **Sweating** is there.
  - **Discussions/Recommendations:** Symptoms appear due to **gastro esophageal reflux (GERD)**. Simple preventive tactics recommended: **Elevate the head of the bed by 6 to 9 inches**. Shared Mayo Clinic link on GERD.
  - **Interventions Implemented:** Reported symptoms.
  - **Results:** BP 92/60 (PR 92), after standing 100/59 (PR 98).
- **Mar 9:**
  - **Symptoms:** **Fever down to 100 F** but still has **body aches, headache, and severe cough**. Cough not subsiding, feels stuck.
  - **Discussions/Recommendations:** Can take **paracetamol six hourly**. Can take **water vapor** if cough feels stuck. Noted patient history linking throat pain and high sugar level as possibly related, substantiated that internal medical inflammations can exacerbate hyperglycemia.
  - **Interventions Implemented:** Fasting blood sugar 109mg/dl, BP 108/74, PR 90. Lunch at 10:30 am. Before lunch took 1/2 Glimepiride 1mg, after lunch took 1/2 tab metformin. 2 hours after lunch sugar 177. After lunch took bisoprolol 2.5 mg around 10 am/10:30 am. After tiffin (puffed rice) took metformin 500 in 1/2tab, 2 hours later sugar 119mg/dl, BP 126/76, PR 96. 2 hours after dinner sugar 120.
- **Mar 12:**
  - **Results:** Patient's weight has come down to 45kg.
- **Mar 15:**
  - **Symptoms:** Indigestion problem since morning. Asked if blood sugar will increase if she eats rice 3 times.
  - **Discussions/Recommendations:** No, sugar depends on what she eats tomorrow. Rice or cereal is not bad for diabetes if consumed in the correct proportion. Advised the Harvard plate proportion for meals.
  - **Interventions Implemented:** Ate rice and vegetables due to indigestion.
- **Mar 16:**
  - **Symptoms:** In the morning, BP 89/57, felt **very weak, eyes getting dark**. After standing, BP 90/49. Eye symptoms: Looks like **episcleritis**, happened quite a few times before. Eye problem used to happen suddenly before it started, eye pressure would increase, slight touch caused eye to move. Feels **heaviness, slight pain, uneasiness to open eyes**, left eye looking small (sentence incomplete). Tennis elbow (lateral epicondylolagia) is a current term for her pain.
  - **Discussions/Recommendations:** Need not take BP medicine in this situation. Episcleritis noted, discussed frequency. Asked if any eye drop would suggest for relief.
  - **Interventions Implemented:** Fasting sugar 105mg/dl, BP 109/65. Took Glimepiride 1mg 1/2tab before lunch. Took Metformin 500 1/2 tab after 2 hours of lunch. After 2 hours sugar 111. Currently on 0.5mg Glimepiride once daily. Post dinner sugar 141mg/dl, took Metformin 250mg. Before lunch took Glimepiride 1mg in 1/2tab. After lunch Metformin 500mg 1/2tab, 2 hours after lunch sugar 123. After tiffin took Metformin 500in 1/2Tab, 2 hours later sugar 148 (tiffin at 3:30 pm). After dinner took Metformin 500 1/2tab, 2 hours later sugar 142.
- **Mar 19:**
  - **Discussions/Recommendations:** Ophthalmologist's clinical findings from Disha noted: Diagnosis of Left corneal opacity with epithelial changes (likely Meesmann's dystrophy) and right cornea with epithelial changes and iris Lisch nodule. Impression: Meesmann's epithelial dystrophy with left corneal opacity (post PTK), right iris Lisch nodule. Rule out Neurofibromatosis Type 1. Confirmed corneal dystrophy diagnosis is entirely clinical. Asked PA to deidentify and share printed prescription.
- **Mar 21:**
  - **Results:** BP 113/57, PR 90.
- **Mar 23:**
  - **Symptoms:** Has not taken BP medicine for last 6 days.
  - **Interventions Implemented:** Fasting blood sugar 94mg/dl. 7 am BP 122/82. Lunch at 10:30. Before lunch took Glimepiride 1mg in 1/2Tab, after lunch took metformin 500 in 1/2 tab. After dinner took metformin 500 in 1/2tab.
  - **Results:** After 2 hours of lunch sugar level 129. After 2 hours of dinner sugar level 96.
- **Mar 25:**
  - **Symptoms:** Patient will reach shortly. Patient feels **very weak with darkness of eyes, slight pain in shoulder and arms**. Thinks the BP machine is not working.
  - **Discussions/Recommendations:** Advised to wait in OPD and will be admitted. Reviewed in OPD. Found **divarication of recti along with a suspected small parietal hernia** at the right hypochondrium on abdominal examination. Planned ultrasound. Review BP instrument and glucometer with ward devices. Ophthalmologist stated corneal dystrophy is not very significant, looks normal, retina normal, field test normal. PTK just ablates tissue, no specimen. Need pre-PTK slit lamp images or visual acuity data. Asked to collect visual acuity data before PTK. Asked to check BP from multiple electronic machines at the same time. Noted breakfast needs to be more substantial.
  - **Interventions Implemented:** Patient reached OPD. BP checked by ward machine (150/90 mmHg vs patient's 103/62, PR 89 at the same time). Patient took roasted makhana in the morning. Ate Idly with chutney and sambar for breakfast. At 7:45 pm BP checked by ward machine (140/70) and own machine (112/69) with 1-2 minute gap.
  - **Results:** Patient's BP 90/60 on OPD review. Differences noted between ward and patient's BP readings. Differences noted between ward and patient's glucometer readings (fasting 122 vs 145, post-lunch 152 vs 172). Later BP readings started matching (110/80 vs 103/64, 110/70 vs 110/69). Glucometer values not matching. Sugar level increasing, 2 hours after dinner 194.

**April 2025:**

- **Apr 8:**
  - **Symptoms:** **Fever has come along with body ache and headache**. Severe body and headache even after taking paracetamol. Abdomen pain looks like previous after taking paracetamol, so stops paracetamol. At night, temperature over 102F with severe pain. Feeling very weak. **Increasing severe muscle cramp**. Again **fever along with headache, nose burning sensation and body ache**.
  - **Discussions/Recommendations:** Advised to take **paracetamol 650**. Monitor temperature 4 hourly. As long as she can eat, she should be fine.
  - **Interventions Implemented:** Stopped BP medicine since last 20 days as per doctor's advice. BP 135/84 (PR 97), after standing 123/81 (PR 91). Patient took paracetamol 650. Took methi mouri water, normal water, raw mango sherbat for muscle cramp. No paracetamol taken yesterday.
  - **Results:** Fever 100F, at night over 102F. No fever on Apr 10, feeling better. Still some discomfort, believes will be fine. Fever 100F again on Apr 11. Muscle cramp felt better after remedies.
- **Apr 11:**
  - **Symptoms:** **Throat completely choked and discomfort for swallowing**.
  - **Discussions/Recommendations:** Paracetamol will work here too. If abdomen pain starts after paracetamol, can avoid medication. Advised to **do gargle with hot water and have black tea**.
- **Apr 12:**
  - **Symptoms:** After taking paracetamol, **abdomen pain starts**. Paracetamol doesn't suit her. **Can't swallow, severe pain in throat**. Feels **constant irritation, watering in right eye**, same symptoms of conjunctivitis.
  - **Discussions/Recommendations:** Advised to avoid medication, take hot water, hot black tea with ginger, boiled daal juice. Try **betadine gargles**. Check for drops last prescribed by Ophthal in her case report.
  - **Interventions Implemented:** Takes paracetamol in the afternoon.
- **Apr 13:**
  - **Symptoms:** Fasting Sugar level 144mg/dl. On the higher side. Past history: **throat pain and high Sugar level is related**. Gargle with Betadine, hot water drinking not working, very painful, can't swallow. Now has 100F. Sugar level 204mg/dl. **Severe throat pain since last night, burning sensation in ears, eyes red and painful, whole face is swollen**. BP and sugar level increasing. Paracetamol not helping. Same problem happened with long train journeys in the past (2015, 2018, 2021, 2023, 2024, 2025), especially March/May. Might be due to long journey and heat wave. Doesn't want to take antibiotics again, feels weak.
  - **Discussions/Recommendations:** Noted fasting sugar is on the higher side. Confirmed glimepiride 0.5mg was taken. Noted the observation linking throat pain and high sugar as good and possibly related, explained inflammation can exacerbate hyperglycemia. Asked when taking breakfast with the tablet. Confirmed she takes glimepiride at usual time (10:30 am). Asked about paracetamol use frequency. Can search case report for similar episode recovering with Paracetamol alone. Asked about glimepiride and metformin timing with high sugar reading. Noted many episodes of fever, eye redness, sore throat in case report for PA to review.
  - **Interventions Implemented:** Fasting Sugar level 144mg/dl. Took glimepiride 0.5 before breakfast yesterday. Not taking breakfast now (9:14 am), takes nuts and black tea, regular breakfast at 10:30 am. BP 130/84 PR 123. Sugar level 204mg/dl. Took paracetamol once after breakfast around 10:30 am. Experienced similar problems after past long journeys, had to take Clavam 625 except in 2025. 2 hours post dinner sugar 148mg/dl, BP 124/83 after taking metformin 500 in half tab.
- **Apr 15:**
  - **Symptoms:** Feeling more better. No taste in mouth, everything feels bitter, sweating a lot. Feeling very weak but improving gradually.
  - **Discussions/Recommendations:** Asked if recent Ophthal evaluation in Mumbai was shared. CR said no. PA said will try but can't deidentify. CR offered to deidentify if sent to their number.
  - **Interventions Implemented:** Patient reported symptoms. Patient had to force herself to work yesterday due to work pressure.
- **Apr 25:**
  - **Symptoms:** Patient and her daughter have been experiencing **a lot of hair fall** (almost 1/3 gone) since last September. Asks about using specific herbal products (Rubodex Hair Oil, Shampoo, Forte Tablet) for hair fall.
  - **Discussions/Recommendations:** Herbal products cannot be reviewed by the experts here, only products with scientific trials. Asked if they changed bath water or checked Vit.D level. Shared information that significant hair fall in another patient was explained by androgen excess. Discussed methods for evaluating and measuring hair loss (daily hair counts, wash test etc.). Shared info on Vitamin D levels and potential for over-testing/overtreatment in India.
  - **Interventions Implemented:** Haven't checked Vit.D level recently, haven't changed bath water since September. Hairfall count 20 today (not significant according to one expert), previously average 3-4. Patient had acne problem previously (cured last year). No male pattern hair distribution. Father and grandfather have baldness.
  - **Results:** Androgen excess suggested as explanation for hair loss.
- **Apr 27:**
  - **Interventions Implemented:** After lunch took metformin 500 in half tab. Lunch included Rice, Dal, bitter gourd pakoda, sabji.
  - **Results:** 2 hours after lunch sugar level 159. BP 113/77, after standing 117/76.

**May 2025:**

- **May 1:**
  - **Symptoms:** Right hand muscles feel very **weak**. Can't apply pressure for pulling or lifting.
  - **Interventions Implemented:** Patient learning muscle strengthening exercise weekly. Using 'Gen Teal' Gel at bedtime for eyes, feels cool and comfortable.
- **May 4:**
  - **Symptoms:** From last 1 week feels **neck pain**.
  - **Discussions/Recommendations:** Neck pain is unrelated to BP. Asked her to elaborate on neck pain using the PaJR history template.
  - **Interventions Implemented:** BP medicine has been stopped since 25 days. Fasting blood sugar 101, BP 97/64. Before breakfast took Glimepiride 1mg in half tab, after breakfast took metformin 500 in half tab. Lunch at 12:49 pm, after took metformin 500 in half tab. Dinner at 6:45 p.m., after took metformin 500 in half tablet.
  - **Results:** 2 hours after breakfast sugar 92. 2 hours after lunch sugar 97.
- **May 5:**
  - **Symptoms:** Since midnight, had **severe nasal congestion, headache, sore throat, intense body ache**. Felt cold even under minimum fan speed. Vision goes **dark whenever stands up**. No fever, **extreme body ache**. Whenever starts full phase work, goes outside, travels by local train, or slight sun exposure, **same symptoms occur (severe throat pain, headache, nose congestion, body ache, eye redness with pain)**. Feels like catching cold easily ('Thanda lege jache'). Pain around eyes started last 1-1.5 month, itching inside eyes since Monday (May 5?). Happening intermittently, alternate eye, almost 2 months, 5 times.
  - **Discussions/Recommendations:** Ophthal opinion: Needs oral antibiotics. Most likely internal hordeolum. Diabetic patients more prone to recurrent infection. Restrain from touching eyes.
- **May 9:**
  - **Symptoms:** Still pain in affected eye. Swelling is there. Hot compress was very painful, so didn't do it. Pain is there, discomfort, feels good if closes eye.
  - **Discussions/Recommendations:** Ophthal opinion needed if recommends antibiotics or face-to-face meeting. Patient Advocate suggests antibiotics needed due to symptoms. Web 2.0 inputs: Share fasting, 2-hour post-meal sugars for next few days till suspected hordeolum subsides. Efficacy of antibiotics doubtful in hordeolum/chalazion over eyelid hygiene and warm compresses based on current evidence ([PubMed], [PMC]). Lack of evidence doesn't imply harm but argues against routine use. Fexofenadine (patient is taking) helps with itching, useful to prevent touching. Detailed plan provided: **Record fasting and 2-hour post-meal glucose values** for several days. **Warm compresses** (10-15 mins, 3-4 times daily, gold standard) and **eyelid hygiene** recommended. Lid massage suggested. Antibiotics not recommended for uncomplicated hordeolum, reserved for cellulitis, immunocompromise, or failure after 1 week of self-care. Follow-up in 5-7 days, refer for I&D if fails to resolve, urgent eval for orbital cellulitis. @PPM4 asks for close-up photo of affected eye. @PPM4 recommends: **Warm moist fomentation**, **Zaha eye ointment (azithromycin 1%)** twice daily, **T. Clavam 625 mg twice daily for 5 days**, along with usual antacid. Ophthal expert @PPM3 analyzes image, likely internal hordeolum manifesting externally, involves meibomian gland. Management implications for internal vs external discussed (warm compresses more critical for internal, topical antibiotic less penetration for internal, oral antibiotics considered for larger/inflammatory internal/resistant). Suggested Action Now: **Begin warm compresses** (5-6 times/day), use **topical antibiotic ointment** (e.g., erythromycin) 2-3 times/day, watch for fluctuation, consider oral antibiotics if worsening/systemic signs, refer if fever/cellulitis/visual disturbance.
  - **Interventions Implemented:** Took fexofenadine 120 at night. Patient reported affected eye is the right one, photo shared. Swelling for 3 days, painful from beginning, warm compress gives relief.
  - **Results:** Completed breakfast at 10 am, took metformin 500 in half tab, 2 hours later sugar 179 mg/dl. Took Glimepiride 1mg in half tab before breakfast. After lunch took metformin 500 in half tab, 2 hours later sugar 92. After dinner took metformin 500 in half tab, 2 hours later sugar 186mg/dl. Took fexofenadine 120 after dinner.
- **May 10:**
  - **Results:** Fasting blood sugar levels 111. Explained sugar difference (lunch 92 due to Glimepiride effect, dinner 186 as effect wore off, still fair control).
- **May 15:**
  - **Symptoms:** Eye condition: **No pain but swelling**. Hot compress was very painful, so didn't do it.
  - **Interventions Implemented:** Completed antibiotic course 3 days before.
- **May 17:**
  - **Symptoms:** **Pain is there**, discomfort, feels good if closes eye.
  - **Discussions/Recommendations:** @PPM3 commented on the role of surgery (Incision and Currettage, Steroid Injection) for resistant stye/hordeolum, considered after 4-6 weeks of failed conservative treatment. Discussed key considerations, timing, risks, and postoperative care. @PPM3 identified the image as likely internal hordeolum manifesting externally and reiterated management suggestions (warm compresses 5-6 times/day, topical antibiotic ointment 2-3 times/day, watch for fluctuation, consider oral antibiotics if worsening).
- **May 18:**
  - **Symptoms:** After breakfast, 2 hours later sugar level 58.
  - **Discussions/Recommendations:** Noted low sugar reading (58), suggested to be careful and **stop taking Glimepiride for a few days**. She can **continue metformin immediately after all three meals** after stopping glimepiride from tomorrow. Advised to monitor response when changing meds.
  - **Interventions Implemented:** Fasting sugar 102mg/dl. Before breakfast took Glimepiride 1mg in half tab (0.5mg). After breakfast took metformin 500 in half tab. Had mango and kheera at 3pm, after that took metformin 500 in half tab. After dinner took metformin 500 in half tab.
  - **Results:** 2 hours after breakfast sugar level 58. Sugar reading 132mg/dl (time unclear). 2 hours after fruits sugar 130. 2 hours after dinner sugar 76.
- **May 19:**
  - **Discussions/Recommendations:** Confirmed to stop glimepiride and continue metformin after meals. Can restart 0.5 mg Glimepiride if necessary after checking today's sugars. Given the low sugar reading (76), advised to stop today's glimepiride and not check sugar for today.
- **May 20:**
  - **Symptoms:** Blister on eye has **burst**. A lot of **pus and blood have come out**.
  - **Interventions Implemented:** Applied Azithromycin ointment.
- **May 25:**
  - **Interventions Implemented:** Glimepiride has been stopped for the last 7 days. Fasting blood sugar level 105. BP 114/72. Patient reported potential food allergy reaction (throat/ear burning after mango). Asked about peanut consumption. Ate breakfast at 8:30 am (roti, chana, pickle).

**May 28, 2025:**

- **Discussions/Recommendations:** Discussion about the therapeutic uncertainty surrounding hordeolum, particularly the efficacy of oral doxycycline. Web 2.0-3.0 inputs noted no solid evidence from RCTs for routine antibiotic use in hordeolum compared to conservative care. Guidelines rely on lower-level evidence and pathophysiologic rationale when RCTs are absent. Practical recommendations reiterated: stepwise approach starting with warm compresses and hygiene, oral antibiotics only if no improvement or systemic signs, I&D for resistant lesions. Patient counseling to emphasize compresses and avoid squeezing.

This timeline provides a structured overview of the patient's health journey, highlighting the interplay between her reported symptoms, the clinical team's assessments and recommendations, the treatments she implemented, and the resulting changes in her condition and measurements.
